# Progress in Iron Oxides Based Nanostructures for Applications in Energy Storage

**DOI:** 10.1186/s11671-021-03594-z

**Published:** 2021-08-31

**Authors:** Linfeng Lv, Mengdi Peng, Leixin Wu, Yixiao Dong, Gongchuan You, Yixue Duan, Wei Yang, Liang He, Xiaoyu Liu

**Affiliations:** 1 School of Mechanical Engineering, Sichuan University, Chengdu, 610065 People’s Republic of China; 2 State Key Laboratory of Advanced Technology for Materials Synthesis and Processing, Wuhan University of Technology, Wuhan, 430070 People’s Republic of China; 3 Med+X Center for Manufacturing, West China Hospital, Sichuan University, Chengdu, 610041 People’s Republic of China

**Keywords:** Iron oxide, Nanostructure, Lithium-ion battery, Anode

## Abstract

The demand for green and efficient energy storage devices in daily life is constantly rising, which is caused by the global environment and energy problems. Lithium-ion batteries (LIBs), an important kind of energy storage devices, are attracting much attention. Graphite is used as LIBs anode, however, its theoretical capacity is low, so it is necessary to develop LIBs anode with higher capacity. Application strategies and research progresses of novel iron oxides and their composites as LIBs anode in recent years are summarized in this review. Herein we enumerate several typical synthesis methods to obtain a variety of iron oxides based nanostructures, such as gas phase deposition, co-precipitation, electrochemical method, etc. For characterization of the iron oxides based nanostructures, especially the in-situ X-ray diffraction and ^57^Fe Mössbauer spectroscopy are elaborated. Furthermore, the electrochemical applications of iron oxides based nanostructures and their composites are discussed and summarized.

**Graphic Abstract** 
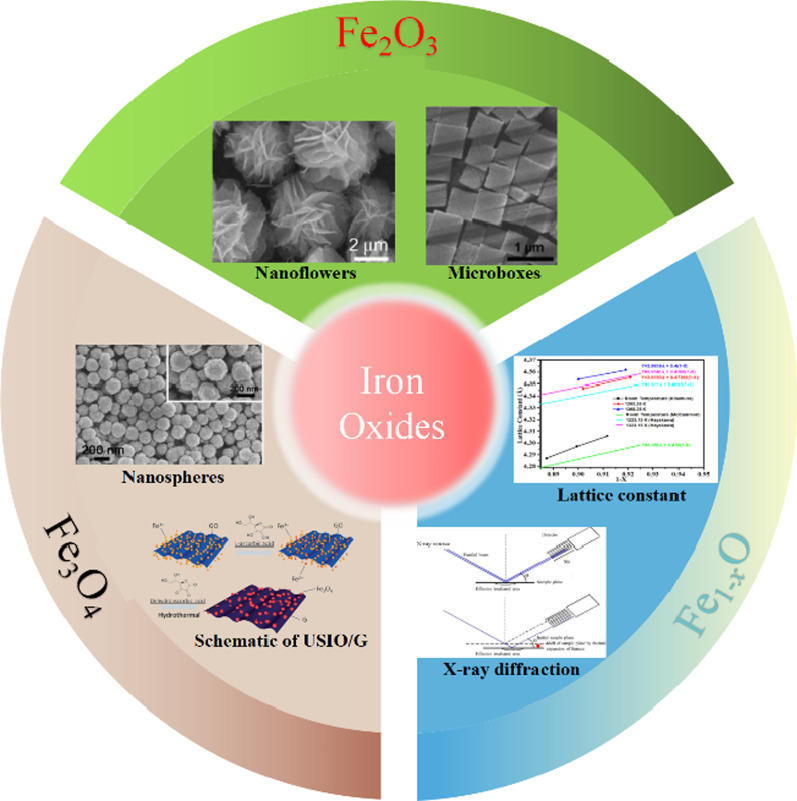

## Introduction

The global energy and environment problems lead to increasing demand for highly efficient green energy, e.g., solar energy, fuel cells, lithium-ion batteries (LIBs) and thermoelectric module, etc. [1–11]. Research and development of high-performance and low-cost energy storage system is an important solution for these problems. Among the energy storage devices with wide applications, LIBs are an important candidates for highly effective energy storage system [12–24]. However, the current commercial graphite anode has the limitations, e.g., a relatively low theoretical capacity (372 mA h g^−1^), and some electrochemically active materials are proposed to be applied in LIBs [25–29]. In 2000, Poizot et al. [30] reported that the transition metal oxides (TMOs) are considered as a kind of important anodes in LIBs since their high theoretical capacities 2–3 times that of graphite. Therefore, TMOs and related composites are attracting much attention as LIBs anode. During lithium insertion/extraction process, TMOs have the following reaction [31].

1 $${\text{M}}_{x} {\text{O}}_{y} + 2y{\text{e}}^{ - } + 2y{\text{Li}}^{ + } \leftrightarrow x{\text{M}}^{0} + y{\text{Li}}_{2} {\text{O}}$$

where, M represent Ni, Cu, Fe, Co, etc. During the lithium insertion process, these oxides are reduced by lithium, and the composite consisting of metallic clusters dispersed in a matrix of amorphous Li_2_O is formed [30, 31].

Among TMOs, iron oxides based anodes are one kind of excellent candidates with great potential in LIBs since they possess such advantages, e.g., abundance, low cost and nontoxicity [32, 33]. However, as similar as other TMOs, iron oxides serving as LIBs anode have two critical issues. One is the large irreversible capacity, caused by decomposition of electrolyte and formation of solid electrolyte interface (SEI) layer in the 1st discharge process. Additionally, the formation of Fe and Li_2_O is thermodynamically feasible, and the extraction of lithium ion (Li^+^) from Li_2_O is thermodynamically instable [34], since a part of Li^+^ cannot be extracted from Li_2_O formed in the 1st discharge process. This also results in partial irreversible capacity. Another issue is their low cycling stability mainly resulted from a large volume variation and severe aggregation of Fe in insertion/deinsertion of Li^+^, leading to pulverization of electrodes and rapid decay in capacity [3]. For solution to these problems, much effort is focusing on overcoming these issues and some highly effective approaches are proposed. To the best of our knowledge, one highly-effective strategy is nanostructuring of iron oxides [3, 35]. For some unique nanostructures, the strain and volume variation resulted from insertion/deinsertion of Li^+^ will be inhibited at large extent, also the Li^+^ can be diffused in electrodes easily, leading to significantly improved electrochemical performance of the anode [11]. Furthermore, carbonaceous materials, e.g., carbon fibers (CFs), carbon nanotubes (CNTs), graphene, and pyrolyzed carbon, etc. are introduced for compositing with iron oxides [36–38]. The volume variation of composite electrodes in charge/discharge can be buffered by these carbonaceous materials with unique structures, hence the electronic contact and the cycling stability of iron oxides nanostructures are increased.

In this review, the recently developed strategies and important research updates on the iron oxides (Fe_1−*x*_O, Fe_2_O_3_, Fe_3_O_4_) based nanostructures with applications in LIBs and supercapacitors are elaborated and summarized. Specifically, we concentrated on the iron oxides based nanostructure’s synthesis and design, as well as their electrochemical performance.

## Wustite (Fe_1−***x***_O)

Wustite (Fe_1−*x*_O) is a non-stoichiometric compound with 0 < *x* < 0.0464 [11]. It has the rocksalt cubic structure, and its lattice constant is ~ 4.330 Å [11]. Fe_1−*x*_O, compared with Fe_2_O_3_ and Fe_3_O_4_, has less applications in energy storage since its relatively low specific capacity and metastable phase below 843.15 K which tends to decompose into Fe and Fe_3_O_4_. However, Fe_1−*x*_O, a highly promising anode for LIBs, has a higher electrical conductivity than those of Fe_2_O_3_ and Fe_3_O_4_.

### Synthesis and Characterization

As far as we know, the compositing of TMOs with high-performance coverage has great potential for high-performance LIBs anode [7]. In LIBs, the electrochemical mechanism of wustite anode is described as the following equation [8].

2 $${\text{FeO}} + 2{\text{Li}}^{ + } + 2{\text{e}}^{ - } \leftrightarrow {\text{Fe}} + {\text{Li}}_{2} {\text{O }}$$

FeO/C composites were synthesized using a facile method by Gao et al. [31]. In their synthesis, α-Fe_2_O_3_ particles with size of 30–120 nm were mixed with acetylene black (AB) of different percentages, and a uniform mixture was obtained by ball milling. Then, the mixture of α-Fe_2_O_3_ and AB was carbonthermally reduced at 800 °C for 10 h in N_2_ atmosphere to obtain uniform FeO/C composites. The FeO/C composite possesses much higher cycling stability than that of Fe_2_O_3_/AB mixture. When the content of AB is 50 wt.%, the capacity of FeO/C composite is 511 mA h g^−1^, higher than 396 mA h g^−1^ of Fe_2_O_3_/AB. Besides, the capacity retention after 50 cycles is > 96%, obviously 70–80% higher than that of Fe_2_O_3_/AB. It is believable that the superior electrochemical performance of FeO/C composite should be resulted from its higher electrical conductivity, resulted from strengthened connection of the FeO and AB particles after carbonthermal reduction.

**Fig. 1:**
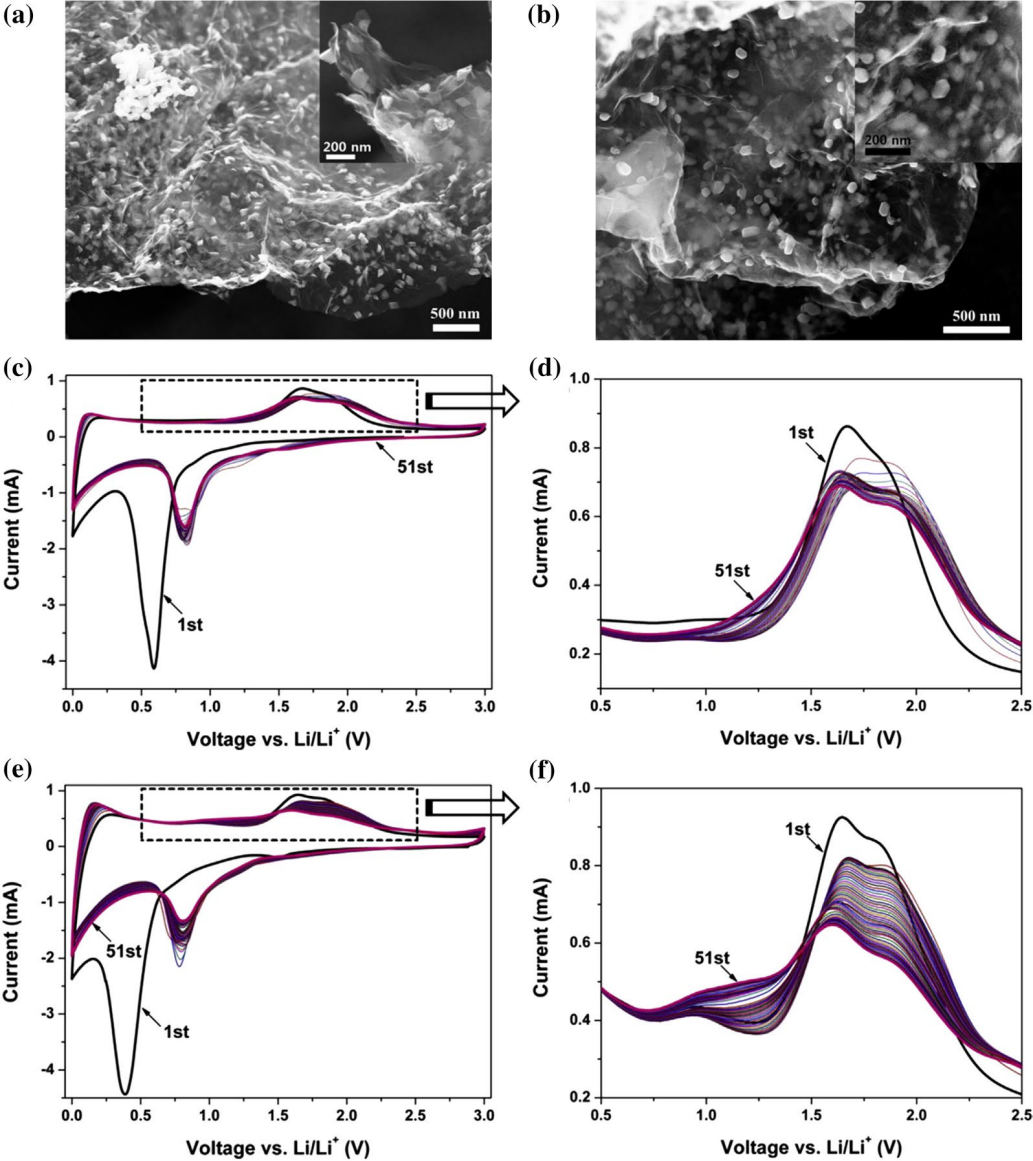
SEM images of **a** Fe_2_O_3_/graphene and **b** K-FeO/graphene, and the inset in (**a**, **b**) are their magnified images, respectively. Repeated cyclic voltammograms of **c**, **d** the Fe_2_O_3_/graphene electrode and **e**, **f** the K-FeO/graphene electrode. Two electrodes were continually scanned for the 51st cycles with a potential sweep rate of 0.5 mV s^−1^. Reprinted with Permission from [11]. Copyright, Elsevier B.V

Afterward, in 2016, Jung et al. [11] prepared a potassium (K)-FeO/graphene composite as LIBs anode based on K-doped FeO nanoparticles by thermal diffusion of K into Fe_2_O_3_/graphene using polyol reduction. Rhombohedral Fe_2_O_3_ crystals were transformed into FeO crystals (face-centered cubic, FCC), showing a broad d-spacing (5.2 Å) of (111) crystal planes, by calcination of K-doped Fe_2_O_3_/graphene. Comparing with previously studied Fe_2_O_3_/graphene composite [11], the K-FeO/graphene showed a discharge capacity of 1776 mA h g^−1^ with high cycling stability during 50 cycles at a current density of 100 mA g^−1^, whereas Fe_2_O_3_/graphene delivered a discharge capacity of 1569 mA h g^−1^. Even at a high current density of 18.56 A g^−1^, the capacity of K-FeO/graphene remained at 851 mA h g^−1^ after 800 cycles. This difference is much larger after the electrodes are cycled longer at a high current density of 18.56 A g^−1^. As shown in Fig. 1, compared with the Fe_2_O_3_/graphene, the K-FeO/graphene anode has unique crystal structure and reaction mechanism. The high discharge capacity of K-FeO/graphene indicates that specific capacity by storage of additional Li^+^ should be contributed from the vacancies and broad d-spacing within Wustite lattices through potassium diffusion into Fe_2_O_3_ lattices.

#### In-Situ X-ray Diffraction

**Fig. 2:**
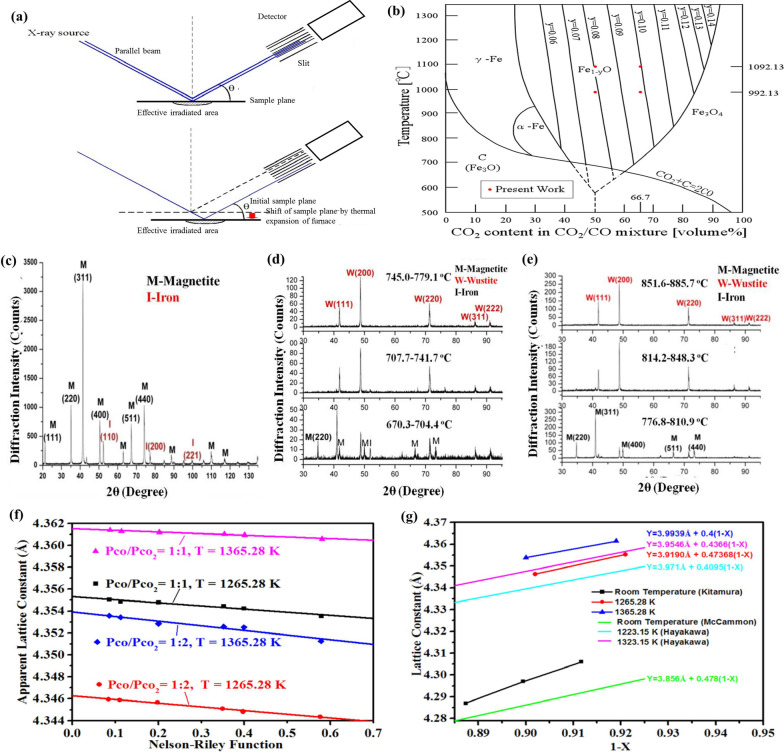
**a** Schematic diagram of in-situ XRD. **b** Equilibrium between Fe, Fe_1−*y*_O, Fe_3_O_4_, CO, CO_2_, and carbon. **c** XRD pattern of initial reactants. **d** XRD pattern of the sample synthesized at Pco/Pco_2_ = 1:1. **e** XRD pattern of the sample synthesized at Pco/Pco_2_ = 1:2. **f** The different NR function-apparent lattice constants of wustite synthesized at different temperatures and Pco/Pco_2_. **g** The results of lattice constant of wustite

By in-situ X-ray diffraction (XRD), the information of real-time structural change during the reaction process of sample, and a large amount of comparable information can be obtained in a short period. It can not only observe the structural change of sample during the synthesis process, it also can be used to detect the corresponding structural change of sample at different temperatures under charge/discharge to a certain potential, which is highly useful for monitoring the actual reaction mechanism of anode/cathode in battery. In addition, our group studied precise knowledge of wustite’s lattice constant that is required for the investigation of its physical and chemical properties at high temperature. The Fe_1−*x*_O was synthesized and characterized by high temperature X-ray diffractometer (RINT2000-TTR, Rigaku Denki Co., Ltd.) with parallel beams (Fig. 2a) for measuring the specific diffraction peaks, and the relation between the composition and lattice constant of wustite at high temperature is also investigated. The synthesis process of wustite with the α-Fe (95 wt.%) and Fe_3_O_4_ (99 wt%) powders as initial materials is a eutectoid reaction (Fig. 2b). This reaction can be proceeded between 843.15 and 1673.15 K at certain Pco/Pco_2_ since the wustite is unstable below 843.15 K, and the reactants are 19% Fe (wt.%) and 81% Fe_3_O_4_ (wt.%). Figure 2c shows the XRD pattern of initial reactants (magnetite and iron). Experiment condition is described in Table 1. After purging Helium (He) gas (25 ml min^−1^) into the furnace of XRD system for 60 min, the reactants were heated with a constant rate of 10 °C min^−1^ until the temperature of samples reached 843.15 K, which were also in He atmosphere with the flow rate of 25 ml min^−1^. Then, the He gas was exhausted, and CO/CO_2_ gas with certain ratio (e.g., 1:1 and 1:2) was purged into the furnace. After the calibration of high temperature by melting Au flakes, the actual temperature of the sample holder could be obtained. From 843.15 K to the desired temperature (1265.28 and 1365.28 K), the heating rate was 2 °C min^−1^ and the XRD was employed to measure the sample for confirmation of the wustite phase. When the sample was reserved at the desired temperature and most of which was wustite phase, the diffraction angles of wustite’s crystal planes were measured during a period of 240–420 min, then the temperature of sample raised for 50 °C and remained at this temperature for 60 min. After these procedures, the temperature of sample decreased to the former desired temperature and the XRD measurement for diffraction angles of crystal planes of wustite was conducted again in a period of 180–240 min.

**Table 1:** Experimental conditions of the XRD measurements

Temperature (K)	Heating rate (°C min^−1^)	Atmosphere	Parameter	Time (min)
293.15–843.15	10	Helium	–	55
843.15–1265.28 (or 1365.28)	2	CO/CO_2_	Step: 0.01°, 5° min^−1^	215 or 265
1265.28 and 1365.28	0	CO/CO_2_	Step: 0.01°, 2° min^−1^ (full scanning), 1° min^−1^ (scanning of 8 crystal planes)	240–420

The lattice constant can be obtained from a linear extrapolating of the apparent lattice constants to zero of this function, that is, 2 Theta = 180°. The diffraction peaks of (111), (200), (220), (311), and (222) crystal planes of wustite are indexed in Fig. 2d, e, and the XRD pattern of the sample is obtained at Pco/Pco_2_ of 1:1 and 1:2, respectively. The relation between apparent lattice constant and Nelson–Riley function under different Pco/Pco_2_ and temperatures is obtained, as shown in Fig. 2f. The straight lines represent the squares fitting to the data. By means of these straight lines, the apparent lattice constants were extrapolated to the zero of Nelson–Riley function. Therefore, as shown in Fig. 2f: the results of the true lattice constants obtained at different temperatures and Pco/Pco_2_ are 4.355 Å (1265.28 K, Pco/Pco_2_ = 1:1), 4.346 Å (1265.28 K, Pco/Pco_2_ = 1:2), 4.362 Å (1365.28 K, Pco/Pco_2_ = 1:1) and 4.354 Å (1365.28 K, Pco/Pco_2_ = 1:2), respectively. As shown in Fig. 2g, the lattice constant increases with the increase of *x* of Fe_1−*x*_O, and the higher the temperature, the larger the lattice constant. The relation between the composition and lattice constant of wustite at high temperature can be obtained as following Eqs. (3) and (4).

3 $${\text{a}}\,\left( { {\AA} } \right) = 3.919 + 0.474\left( {1 - x} \right){ }\left( {1265.28\,{\text{ K}}} \right)$$

4 $${\text{a}}\,\left( { {\AA} } \right) = 3.994 + 0.400\left( {1 - x} \right){ }\left( {1365.28\,{\text{ K}}} \right)$$

#### ^57^Fe Mössbauer Spectroscopy

The ^57^Fe Mössbauer spectroscopy involves properties of the nucleus, including energy level structure of the nucleus and the chemical environment in which the nucleus is located. Hence, it can be applied accordingly to study the valence of atoms, the ionicity of chemical bonds, coordination number, crystal structure, electron density and magnetic properties of sample. The ^57^Fe Mössbauer spectroscopy is widely utilized in the fields of chemistry and materials. Herein, we elaborate the ^57^Fe Mössbauer spectroscopy for characterizing iron oxides. The ^57^Fe Mössbauer spectroscopy is used to distinguish and characterize various iron oxide phases, and to monitor the local environment of Fe atoms in crystal lattice [39, 40].

The hyperfine parameters, such as isomer shift (IS), quadrupole splitting (QS), quadrupole shift (*ɛ*_Q_) and hyperfine magnetic field (*B*_hf_) can be obtained by analyzing the position of the spectral lines in Mössbauer spectrum [41, 42]. The characteristics of the sample can be inferred from the width and asymmetry of the spectral lines. Through temperature and field dependence of the hyperfine parameters also allows deducing valuable parameters.

**Fig. 3:**
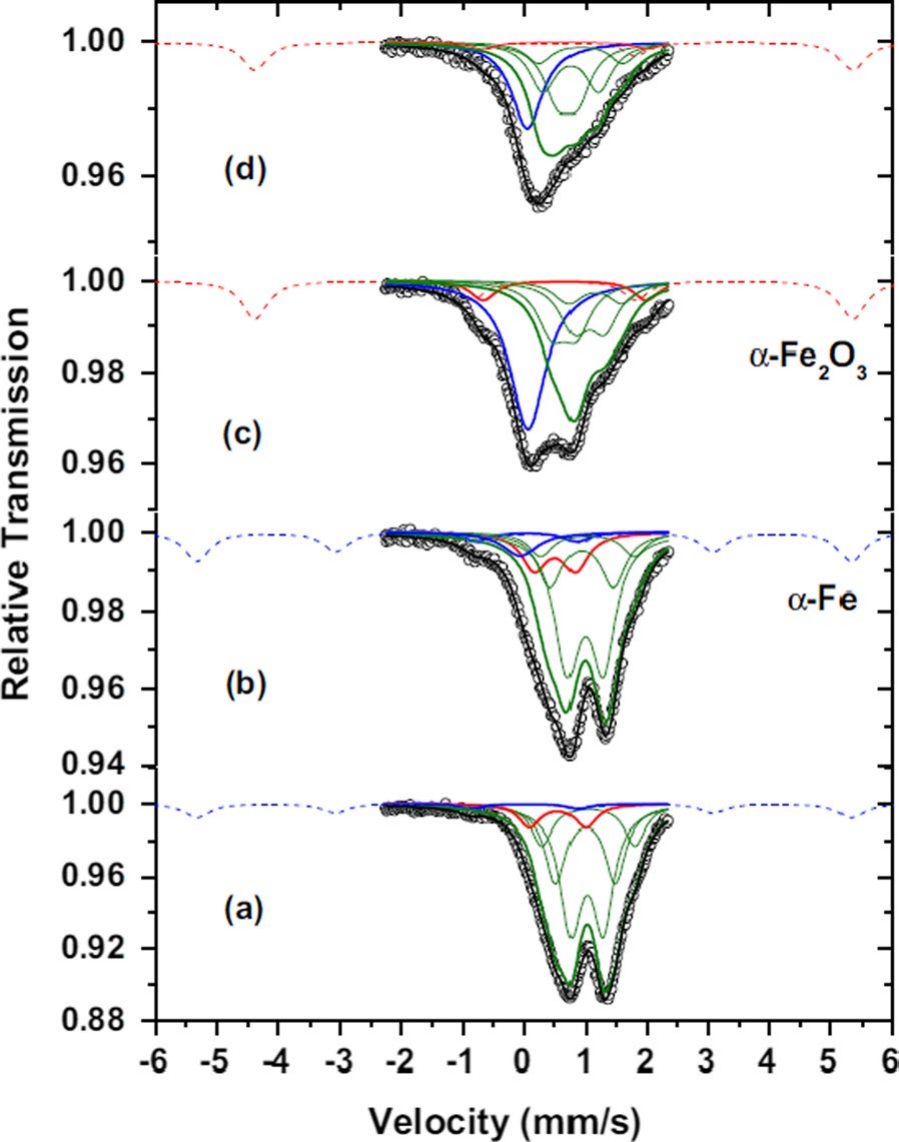
Comparison of ^57^Fe Mössbauer spectra at 300 K of the pristine Fe_1−*x*_O material (**a**), after an uptake of 1 Li (**b**) and the end for discharge at 2.16 Li (**c**). Green contributions are attributed to FeII. The thicker green line is sum of the thinner one corresponding to unreacted Fe_1−*x*_O. Dashed blue line corresponds to the expected increasing contribution of α-Fe from (**a**) to (**c**). In the case of (**c**), magnetic sextet has been slightly shifted as a guide of the eye. Spectrum (**d**) corresponds to the end of the first charge at 0.94 Li with no α-Fe contribution, but the blue singlet nanosized metallic ε-Fe^0^ is still present. Reprinted with Permission from [42]. Copyright, Elsevier Masson SAS

Aldon et al. [42] studied lithium-induced conversion reaction of Fe_1−*x*_O using ^57^Fe Mössbauer spectroscopy. The hyperfine parameters (IS and QS) are rather characteristic of FeII species in antiferromagnetic (TN = 198 K [43]) Fe_1−*x*_O, showing a typical paramagnetic absorption at room temperature (RT). As indicated in the ^57^Fe Mössbauer spectrum (Fig. 3a), there are three broadened doublets with an IS ~ 1 mm s^−1^ and QS ranging from ~ 0.50 to 1.50 mm s^−1^, and their relative areas are corrected from α-Fe magnetic contribution. The absorption intensities are 42, 26 and 15%. A fourth doublet centered at IS ~ 0.55 mm s^−1^, QS ~ 0.90 mm s^−1^ with a relative area of 11% is common for FeII^I^ species, as expected in non-stoichiometric Fe_1−*x*_O. From the FeII/FeII^I^ ratio, the amount of vacancies is estimated as ~ 0.057 ± 0.008, closed to 0.050 by XRD characterization. Finally, the doublet located at QS ~ 1.68 mm s^−1^ and IS ~ 0 mm s^−1^, corresponding to α-Fe, makes a contribution of ~ 6%.

Comparing with Fe_2_O_3_ and Fe_3_O_4_, the specific capacity of Fe_1−*x*_O is lowest. Besides, its synthesis method is more complicated. In most cases, the reduction reaction at high temperature is inevitable [3–6, 9, 11]. As a result, Fe_1−*x*_O is not an ideal LIBs anode comparing with Fe_2_O_3_ or Fe_3_O_4_.

## Fe_2_O_3_ Based Nanostructures

Among these iron oxides, especially Fe_2_O_3_, is attracting many researchers’ attention due to the high theoretical capacity, which can reach 1000 mA h g^−1^ [44]. Additionally, Fe_2_O_3_ has distinctive advantages, such as high resistance to corrosion, low production cost, environmental friendliness, nonflammability, nontoxicity and high natural availability [45]. Due to these excellent properties, Fe_2_O_3_ is highly promising for applications in LIBs anode [46–50]. Herein, a concise overview of recent development about the synthesis, characterization and electrochemical performance of Fe_2_O_3_ based nanostructures is provided.

### Synthesis and Characterization

In last decade, there are enormous efforts for exploring synthetic methods of Fe_2_O_3_ based nanostructures. In this section, we elaborated and summarized synthetic methods of Fe_2_O_3_ based nanostructures, including gas phase deposition [51], solution based method [52], electrochemical method [53], thermal treatment [54] and other methods [55, 56]. Also, we compared different synthesis methods.

Additionally, the ^57^Fe Mössbauer spectroscopy characterization of Fe_2_O_3_ nanostructures is described in details. Since only certain nuclei has resonance absorption, the ^57^Fe Mössbauer spectroscopy is not interfered by other elements. The range of the ^57^Fe Mössbauer spectroscopy affected by extranuclear environment is generally within several nanometers, so it is very suitable for characterizing nanostructure.

#### Gas Phase Deposition

Gas phase deposition is widely applied in synthesis of many thin films and other nanostructures, such as Fe_2_O_3_ and other iron oxides based nanostructures. Chemical vapor deposition (CVD), atomic layer deposition (ALD), physical vapor deposition (PVD), electrolytic deposition and reactive sputtering are typical methods of gas phase deposition [57–60].

ALD is a unique way to synthesize high-crystallinity thin films, and a cheaper route than liquid phase deposition. In ALD process, the chemical reaction of every layer is directly accompanied with the former layer. In this way, only one layer is deposited per reaction cycle. For instance, Lin et al. [51] utilized ALD to deposit a high-quality ultrathin α-Fe_2_O_3_ film on TiSi_2_ nanonets. The 3D self-organized nanoporous thin films were fabricated by Yang et al. [61] through CVD and integrated into a heterogeneous Fe_2_O_3_/Fe_3_C-graphene. As LIBs anode, it’s rate capacity and cyclability can be greatly improved by deposition of this thin film. Cesar et al. [62] deposited thin films of silicon-doped Fe_2_O_3_ dendritic nanostructures by atmospheric pressure CVD (APCVD), which produced Fe_2_O_3_ photoanodes that oxidize water under visible light with unprecedented efficiency. The dendritic α-Fe_2_O_3_ nanostructures showed a macroscopic surface area of 0.5 cm^2^ [62]. The vertically aligned α-Fe_2_O_3_ nanorods array is grown on a silicon substrate via metal–organic CVD (MOCVD) by Wu et al. [63]. What’s more, Jia et al. [64] utilized a radio frequency sputtering deposition to fabricate α-Fe_2_O_3_ ultrathin films.

Although gas phase deposition is capable of preparing high-quality Fe_2_O_3_ based nanostructures, it also has disadvantages. For example, APCVD and MOCVD have high toxicity and flammability in the process of precursors.

#### Solution Based Synthetic Method

**Fig. 4:**
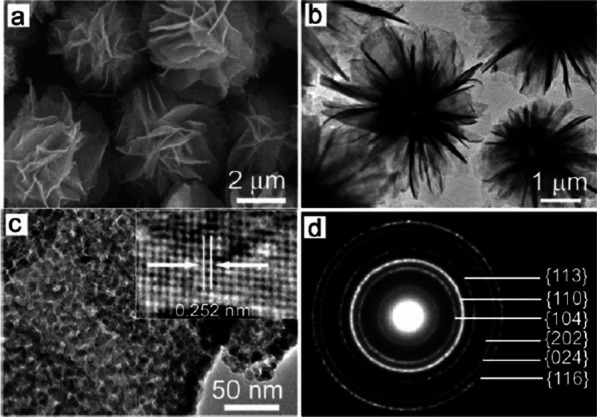
**a** SEM and **b** TEM images of the as-obtained α-Fe_2_O_3_. **c** High-magnification TEM image of the petal of the flowerlike structure of the as-obtained α-Fe_2_O_3_. **d** SAED pattern of the obtained α-Fe_2_O_3_. Reprinted with Permission from [65]. Copyright, Wiley-VCH

**Fig. 5:**
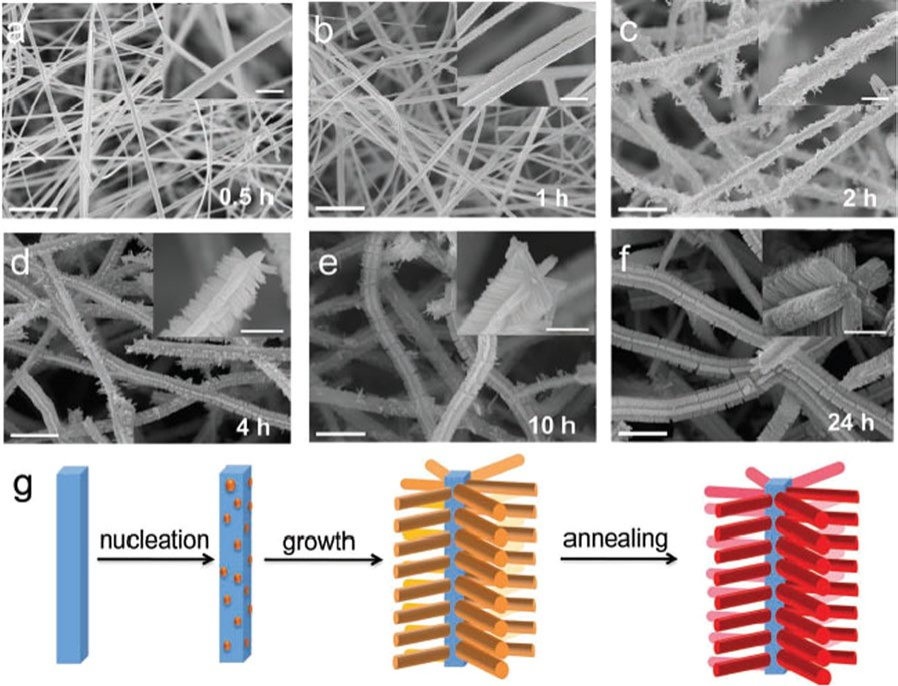
**a**–**f** SEM images of the products (after annealing) at various reaction stages by setting the reaction time. The insets are the corresponding magnified SEM images. The scale bars in the figures and insets are 2 μm and 500 nm, respectively. **g** Schematic of the formation process of the hierarchically assembled α-Fe_2_O_3_/SnO_2_ nanocomposite. Reprinted with Permission from [74]. Copyright, Wiley-VCH

The solution based synthetic method is common and facile to fabricate Fe_2_O_3_ and other iron oxides. Fe_2_O_3_ with various morphologies, such as nanoflowers [65], nanospheres [66], nanoparticles [67], nanorods [68], nanotubes [14], nanorings [69], nanobelts [70], nanoflakes [71], nanowires [72], nanofibers [73], and microboxes [54], were synthesized by solution based method, e.g., hydrothermal, solvothermal and sol–gel approaches. These methods are highly facile and available. Zhong et al. [65] used a solvothermal method to fabricate Fe_2_O_3_ nanoflowers (Fig. 4) via an ethylene glycol-mediated self-assembly process. Vayssieres et al. [52] reported the growth of porous Fe_2_O_3_ nanorods array on fluorine doped tin oxide (FTO) conducting glass by a hydrothermal process. By hydrothermal growth of α-Fe_2_O_3_ precursor on SnO_2_ nanowire stems, a novel six-fold-symmetry branched α-Fe_2_O_3_/SnO_2_ heterostructure (Fig. 5) was synthesized [74]. There is another facile and economical technique, sol–gel method, for synthesizing Fe_2_O_3_ nanostructures. Woo et al. [68] utilized a sol–gel method to obtain α-Fe_2_O_3_ nanorods by reaction of ubiquitous Fe^3+^ in reverse micelles. The nanorods obtained by this mechanism have low dimensionality and high surface area, which can be extended to magnetite and wustite.

#### Electrochemical Method

Electrochemical method is utilized to synthesize Fe_2_O_3_ nanostructures, e.g., the electrochemical deposition is applied in fabricating Fe_2_O_3_ nanoparticles [53]. Through anodization of iron foil in ethylene glycol electrolyte solution containing deionized (DI) water and NH_4_F at a voltage of 30–60 V, α-Fe_2_O_3_ nanotubes array was obtained [75]. In addition, electrochemical anodization is employed to synthesize Fe_2_O_3_ nanotubes array [76]. Mao et al. [77] reported the synthesis of Fe_2_O_3_ array using electrochemical deposition. In their research work, iron was deposited into AAO template channels by electrochemical deposition, then the AAO template was removed by NaOH solution, and finally the iron nanorods array was converted to Fe_2_O_3_ array. The characteristic of this research is that, by changing the duration of deposition, the length of Fe_2_O_3_ nanorods can be finely controlled.

#### Thermal Treatment

**Fig. 6:**
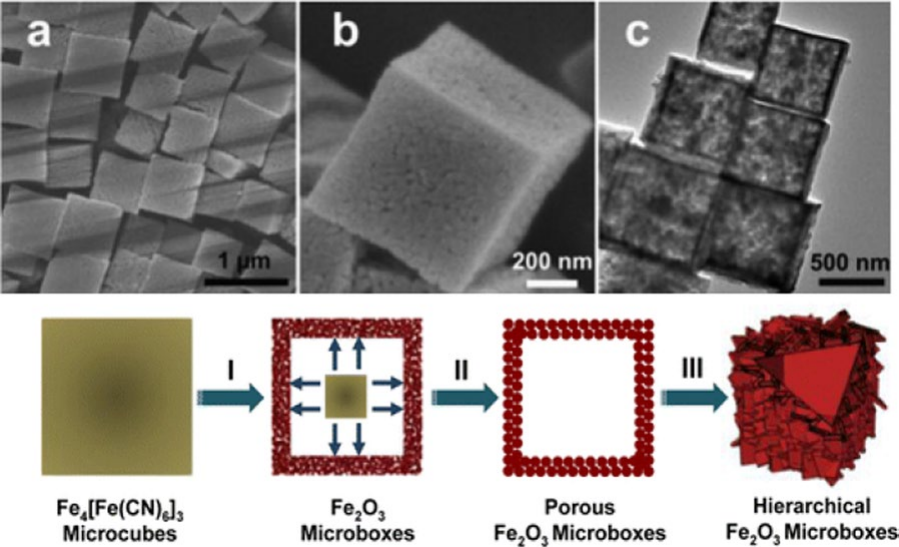
**a**, **b** FESEM and **c** TEM images of hollow Fe_2_O_3_ microboxes obtained at 350 °C. **d** Schematic illustration of the formation of hollow Fe_2_O_3_ microboxes and the evolution of the shell structure with the increasing calcination temperature. Reprinted with Permission from [54]. Copyright, American Chemical Society

The thermal treatment for synthesizing Fe_2_O_3_ involves two significant approaches, thermal oxidation and thermal pyrolysis. For example, Zhang et al. [54] prepared Fe_2_O_3_ microboxes (Fig. 6) via thermally induced oxidative decomposition of prussian blue (PB) microcubes at 350–650 °C. The solid-state approach will provide a more facile way for large-scale synthesis of uniform anisotropic hollow structures in comparison with the widely used solution based method. Fe_2_O_3_ with different morphologies were prepared via commanding thermal oxidation parameters. α-Fe_2_O_3_ nanostructures by facile thermal treatment of iron based precursors are proposed. For instance, Rao and Zheng [71] utilized heat treatment to prepare densely aligned α-Fe_2_O_3_ nanoflakes array.

Thermal pyrolysis is another common method to deposit Fe_2_O_3_ thin films. Duret et al. [78] applied this method to obtain mesoscopic α-Fe_2_O_3_ leaflet films through ultrasonic spray pyrolysis, while the fabricated films have higher photoactivity than those fabricated by conventional spray pyrolysis techniques. Also, the synthesis of α-Fe_2_O_3_ nanoflakes, nanoflowers, nanowires and nanorods array via vapor phase deposition, liquid phase deposition and thermal treatment is reported [63, 71, 72, 79].

#### Other Methods

In 2016, Guivar et al. [55] compounded vacancy ordered maghemite (γ-Fe_2_O_3_) nanoparticles functionalized with nanohydroxyapatite (nanoHAp), using a typical co-precipitation chemical route. Remarkably, the γ-Fe_2_O_3_ functionalized with nanoHAp labeled as γ-Fe_2_O_3_@HAp is formed without any thermal process as calcination.

There are many ways to synthesize Fe_2_O_3_, but most of them are not environmentally friendly. In 2019, Bashir et al. [56] developed an eco-friendly method to obtain α-Fe_2_O_3_ nanoparticles, using Persea Americana seeds extract. They used two different precursors to prepare two samples of α-Fe_2_O_3_, one sample (A) prepared from Fe(NO_3_)_3_·9H_2_O, and another sample (B) prepared FeCl_3_·9H_2_O.

**Fig. 7:**
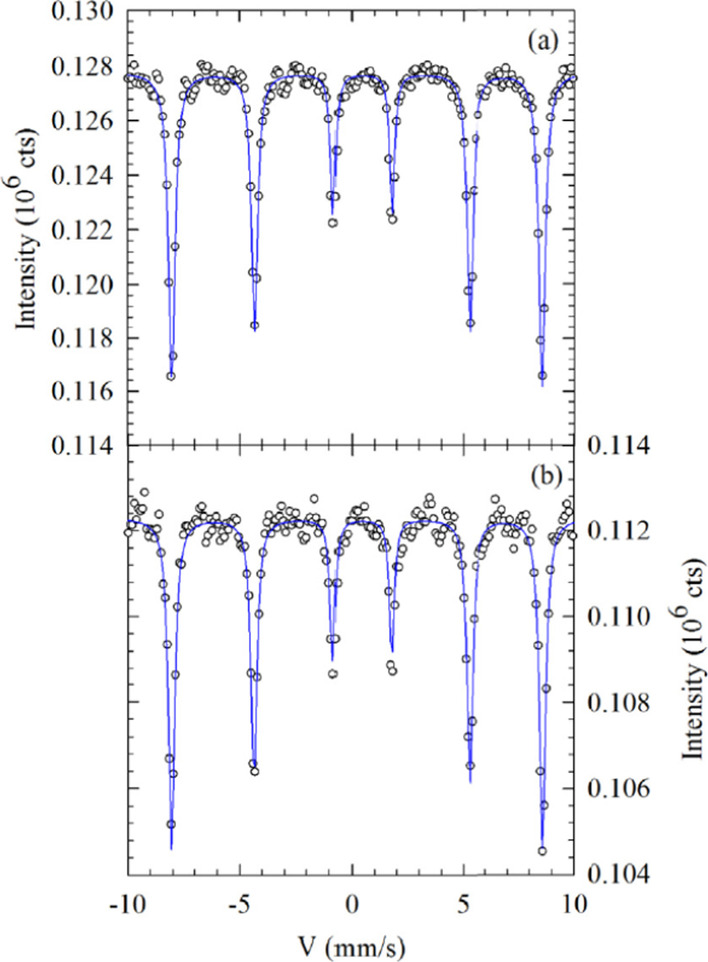
^57^Fe Mössbauer spectra recorded at room temperature for α-Fe_2_O_3_ nanoparticles. **a** sample A (ferric nitrate as a precursor) and **b** sample B (ferric chloride as a precursor). Reprinted with Permission from [56]. Copyright, American Chemical Society

The ^57^Fe Mössbauer spectra of samples A and B recorded at 300 K (room temperature (RT)) are presented in Fig. 7. The ^57^Fe Mössbauer spectroscopy is a very valuable technique for exploring the local magnetic behavior and oxidation state of iron atoms in a particular matrix [80]. Both samples revealed magnetic ordering, and displayed only single sextet indicating magnetically ordered state. Table 2 shows ^57^Fe extracted Mössbauer parameters at RT, which lists IS, *ɛ*_Q_ and *B*_hf_. Both samples’ *B*_hf_ above 51 T are related to α-Fe_2_O_3_ [81]. Furthermore, the values of *ɛ*_Q_ is also consistent with α-Fe_2_O_3_. Both quadrupole interactions indicate Fe as Fe^3+^ since the observed IS of 0.3653 mm/s and 0.3754 mm/s for the samples A and B, respectively, are typical for Fe^3+^ [82]. Therefore, the negative values of quadrupole splitting indicate the weak ferromagnetic property of the samples A and B, the characteristic of pure α-Fe_2_O_3_ phase.

**Table 2:** Mössbauer parameters IS, (ɛ_Q_), B_hf_ and relative area for samples A and B (Reprinted with Permission from [56]. Copyright, American Chemical Society.)

Sample	IS (mm s^−1^)	*ɛ*_Q_ (mm s^−1^)	*B*_hf_ (T)	Relative area (%)
A	0.36 (1)	− 0.095 (1)	51.6 (4)	100
B	0.37 (1)	− 0.11 (1)	51.4 (3)	100

### Electrochemical Performance

**Fig. 8:**
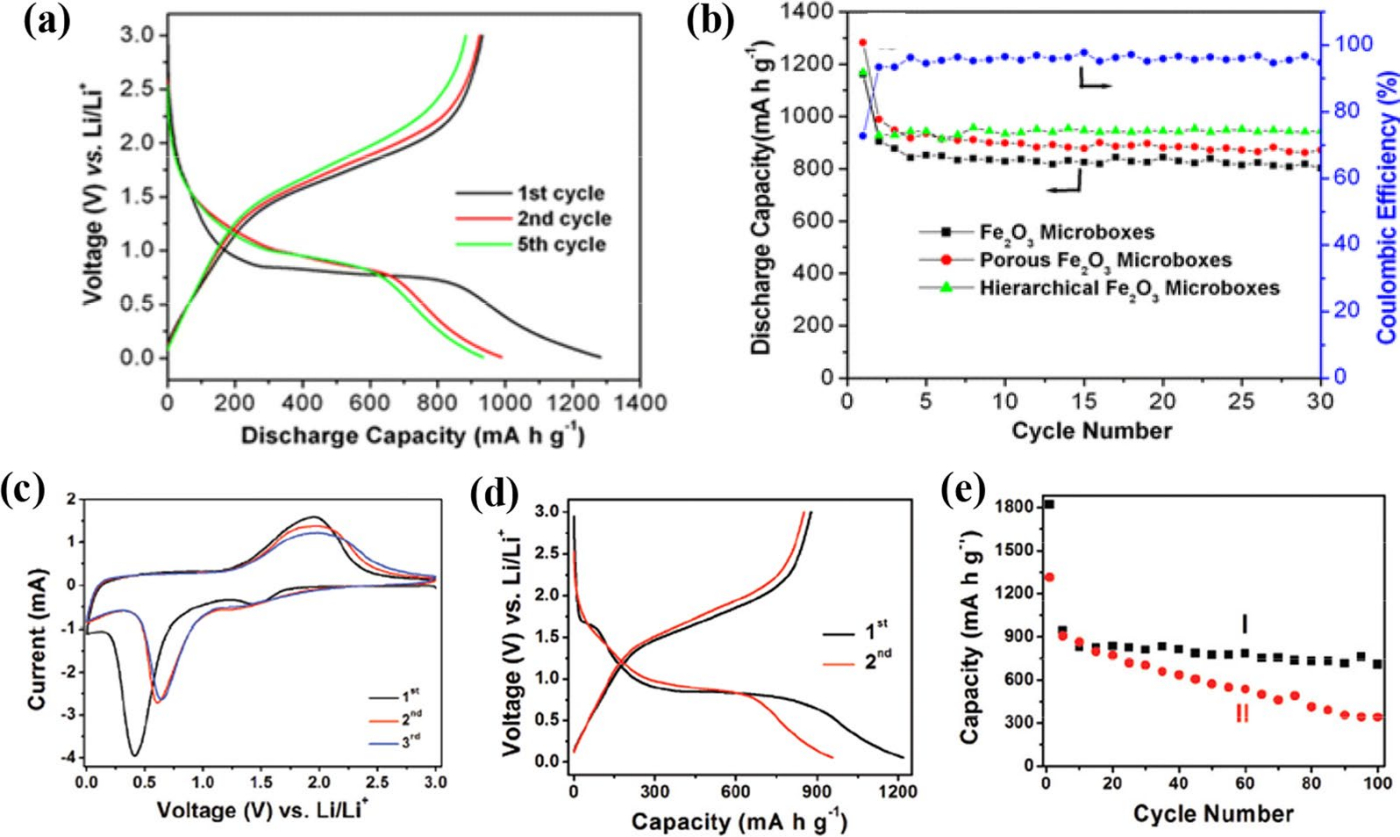
**a** Discharge − charge voltage profiles of porous Fe_2_O_3_ microboxes obtained at 550 °C. **b** Cycling performance of Fe_2_O_3_ microboxes (350 °C), porous Fe_2_O_3_ microboxes (550 °C), and hierarchical Fe_2_O_3_ microboxes (650 °C) and Coulombic efficiency of porous Fe_2_O_3_ microboxes (550 °C) over the voltage range 0.01 − 3.0 V vs. Li/Li^+^ at the same current density of 200 mA g^-1^. (**a**, **b** Reprinted with Permission from [54]. Copyright, American Chemical Society). Electrochemical measurements of the sample. **c** CVs between 5 mV and 3 V at a scan rate of 0.5 mV s^−1^. **d** Charge–discharge voltage profiles. **e** Comparative cycling performance of (I) the as-prepared α-Fe_2_O_3_ hollow spheres and (II) α-Fe_2_O_3_ microparticles. All the galvanostatic tests are performed at a constant current rate of 200 mA g^−1^ between 0.05 and 3 V. **c**–**e** Reprinted with Permission from [85]. Copyright, American Chemical Society

The charge/discharge cycling at the voltage window of 0.005–3.0 V (vs. Li^+^) under a current density of 200 mA g^−1^ at RT is shown in Fig. 8a. During the initial discharge process, there is an obvious voltage platform of ~ 0.75 V, and it gradually moved to a voltage plateau of ~ 1.0 V, and remain stable in the second and fifth cycles. Meanwhile, an ambiguous plateau was observed at ~ 1.8 V in the charge process. The first discharge profile qualitatively resembles the results by Larcher et al. [83] and Morales et al. [84] on nanoparticle Fe_2_O_3_, and Wang et al. [14] on Fe_2_O_3_ nanotubes. The cycling performance of three samples (hierarchical Fe_2_O_3_ microboxes, Fe_2_O_3_ microboxes and porous Fe_2_O_3_ microboxes) is discribed in Fig. 8b. After 30 cycles, hierarchical Fe_2_O_3_ microboxes exhibit the highest reversible capacity of 945 mA h g^−1^, follow by 872 mA h g^−1^ for porous Fe_2_O_3_ microboxes, and finally 802 mA h g^−1^ for Fe_2_O_3_ microboxes. The results demonstrated that three samples display excellent cycling stability, and the morphology of nanostructured Fe_2_O_3_ plays a significant role in determining the discharge characteristics.

Cyclic voltammogram (CV) is used to characterize the cells with α-Fe_2_O_3_ nanoflakes anode in the 0.005–3.0 V under a slow scan rate (at RT). Li metal is used as the counter and reference electrodes [20], consistent with previously reported results [85]. CV curves of α-Fe_2_O_3_ hollow spheres between 5 mV and 3 V at a scan rate of 5 mV s^−1^ are presented in Fig. 8c. There are apparent redox current peaks, and demonstrate good reversibility of electrochemical reaction. As shown in Fig. 8d, a distinct voltage plateau can be discovered at ~ 0.75 V, consistent with CV curves. The charge–discharge voltage profiles reflect the lithium storage capacity of α-Fe_2_O_3_. The first cyclic discharge capacity and the charge capacity is 1219 mA h g^−1^ and 877 mA h g^−1^ respectively, which lead to a relatively low irreversible capacity loss of 28%. In the second cycle, the Coulombic efficiency increased quickly to 89%. Cycling performance of two samples are demonstrated in Fig. 8e. The sample (I) exhibits excellent cyclic capacity retention from the second cycle onward. After 100 cycles of charge/discharge, the reversible capacity is still as high as 710 mA h g^−1^. Compared with α-Fe_2_O_3_ microparticles, the unique hierarchical α-Fe_2_O_3_ hollow spheres apparently have enhanced Li storage performance, and a more stable cycling capacity retention and a higher reversible capacity are realized. This superior performance can be attributed to the thin nanosheet subunits that provide rapid and efficient transport of Li^+^, as well as the unique hollow interior that allows the material to effectively buffer the stress generated during charge/discharge process.

Iron oxides are cheap, abundant and environmentally compatible, and Fe_2_O_3_ has excellent electrochemical performance. Some of the above studies have shown that Fe_2_O_3_ based nanostructures can be an alternative anode to replace the presently used graphite in LIBs. The nanostructured Fe_2_O_3_ has great potential in LIBs anode [86–95]. Recently, several studies about Fe_2_O_3_ anode in asymmetric supercapacitors are reported [96–100]. However, low surface area and poor electrical conductivity are still two critical issues limiting the specific capacitance and power density of Fe_2_O_3_. For solutions of these problems, CNTs and CNFs are regarded as conductive matrices to load Fe_2_O_3_ nanoparticles for realizing improved performance [101]. Table 3 summarizes some typical Fe_2_O_3_ based nanostructures with their synthesis and electrochemical performance.

**Table 3:** A summary of electrochemical performance of Fe_2_O_3_ based nanostructures

Active electrode	Synthesis	Electrochemical performance	References
Single-crystalline α -Fe_2_O_3_ nanosheets on conductive substrates	Thermal heating	700 mA h g^−1^ after 80 cycles at 65 mA g ^−1^	[20]
Uniform single-crystalline α-Fe_2_O_3_ nanodiscs	Controlled oxalic acid etching process	662 mA h g^−1^ after 100 cycles at 200 mA g^−1^	[102]
Single-crystalline α-Fe_2_O_3_ nanosheets grown directly on Ni foam	Template-free growth	518 mA h g^−1^ after 50 cycles at 0.1 C	[103]
Hierarchical α-Fe_2_O_3_ hollow spheres with sheet-like subunits	Quasimicroemulsion-templated hydrothermal reaction	710 mA h g^−1^ after 100 cycles at 200 mA g^−1^	[85]
Hierarchical Fe_2_O_3_ microboxes	Annealing prussian blue (PB) microcubes	945 mA h g^−1^ after 30 cycles at 200 mA g^−1^	[54]
Carbon-coated α-Fe_2_O_3_ hollow nanohorns grafted on CNT backbones	Direct growth and thermal transformation of β-FeOOH nanospindles on CNTs, followed by carbon nano-coating	800 mA h g^−1^ after 100 cycles at 500 mA g^−1^	[104]
α-Fe_2_O_3_ nanowires	Low-temperature CVD	456 mA h g^−1^ after 100 cycles at 0.1 C	[105]

## Fe_3_O_4_ Based Nanostructures

Magnetite (Fe_3_O_4_) and Fe_3_O_4_ based nanostructure composites with pseudocapacitance, high theoretical capacity, environmental friendliness and low cost, are extensively applied to electromagnetic wave absorption [106], LIBs [3, 12, 15], biotechnological devices [107, 108], and supercapacitor [4, 109]. Fe_3_O_4_ nanoparticles is one of the high-performance anodes in electrochemical devices. Unfortunately, Fe_3_O_4_ nanostructures still face some problems, such as a severe volume variation (~ 200%) during the insertion and extraction of Li^+^ and relatively low electrical conductivity, posing negative influence on the cycling stability [19, 110, 111]. From research results, we found that the structure and morphology of Fe_3_O_4_ have a high influence on the electrochemical performance of Fe_3_O_4_ and Fe_3_O_4_ based composites.

### Synthesis and Characterization

Recently, nanostructure engineering is demonstrated as a highly-effective approach to obtain improved electrochemical performance of Fe_3_O_4_ and Fe_3_O_4_ based composites. Therefore, various nanostructures including 0D nanoparticles [112], 1D nanorods/wires [113, 114], 2D nanoflakes/sheets [115, 116], 3D hierarchical/porous architectures [117, 118], and hybrid nanostructures of iron oxides [16] are proposed. The electrochemical performance of Fe_3_O_4_ nanostructures can be optimized by rational design of their morphology, composition, porosity and surface characteristics.

Solution phase synthetic method is a facile and rapid way to obtain Fe_3_O_4_ based nanostructures, because of the associative advantages, such as low synthesis temperature (always below 250 °C), easy control of morphology via adjusting hydrothermal conditions (e.g., PH, density of reactant and dosage of active agent, etc.). Solution phase synthetic method includes solvothermal synthesis [119], thermolysis [120], co-precipitation [121], sol–gel process [122, 123], micro-emulsion [124], etc. Simultaneously we compare the pros and cons of these methods.

#### Solvothermal Synthesis

Solvothermal synthesis, which reacts in a special closed reaction vessel, is one commonly used methods for synthesizing Fe_3_O_4_. In a hermetic environment, it is a facile method using aqueous solution as reaction medium at high temperature and high-pressure hermetic environment. Fe_3_O_4_ nanostructures with various morphologies (0D, 1D, 2D and 3D) were synthesized applying this approach.

An et al. [119] obtained the Fe_3_O_4_/graphene nanowires by solvothermal synthesis and calcination with FeCl_3_, NH_4_VO_3_ and graphene as precursors. Phase transition of Fe_3_O_4_/VO_*x*_ (FVO) after annealing was confirmed by XRD. For the XRD pattern of sample without annealing, all diffraction peaks of FVO and graphene decorated FVO correspond to FeVO_4_·1.1H_2_O. For the XRD pattern of sample after annealing, there are no peaks of any vanadium oxides. And the inductive coupled high frequency plasma (ICP) result indicated that the molarity ratio of Fe and V is ~ 0.94:1, confirming the existence of amorphous vanadium oxide.

Mu et al. [125] reported dispersed Fe_3_O_4_ nanosheets on carbon nanofiber by combing the electrospinning and solvothermal process. In this work, Fe_3_O_4_ nanosheets are uniformly attached on the surface of carbon nanofiber with the diameter of about 500 nm.

Fe_3_O_4_ nanoparticle with high specific surface area via FeCl_3_ and organic solvent ethanolamine (ETA) as precursors is reported by Wang et al. [126]. In this preparation, ETA is critical factor for compounding Fe_3_O_4_ nanoparticles with high specific surface area, and Fe^3+^ is gradually reduced to Fe^2+^ by ETA during dissolution process, demonstrating that Fe^2+^ increased as the increase of ultrasonication time. The ratio of ETA and FeCl_3_ has a large impact on the nanoscale grain size and specific surface area of Fe_3_O_4_. And the results showed that the grain size of 20–40 nm is achieved with 60 mL ETA and 6 mmol FeCl_3_. When the amount of ETA is 80 mL, smaller nanoparticles (5–10 nm) are obtained.

**Fig. 9:**
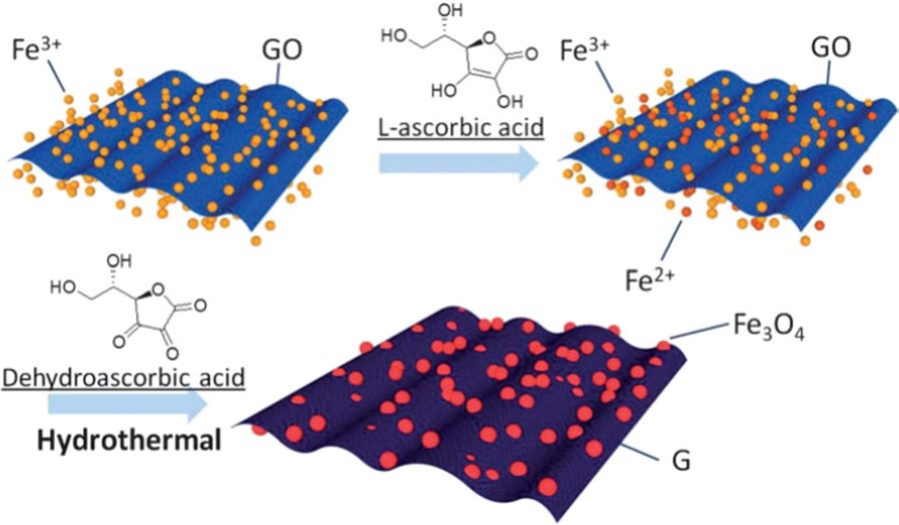
Schematic illustration of the formation process of USIO/G. Reprinted with Permission from [127]. Copyright, Royal Society of Chemistry

Another representative work is reported by Chen et al. [127], in which graphene nanosheets decorated with Fe_3_O_4_ nanoparticles (USIO/G) were synthesized using a facile solvothermal process. For the synthesis of USIO composite decorated with reduced graphene oxide (RGO), is used FeCl_3_·H_2_O as precursor, then NaHCO_3_ and L-ascorbic acid were added to form USIO/G. In this process, L-ascorbic acid was oxidized to dehydroascorbic acid (DHAA) by some of Fe^3+^, which were reduced to Fe^2+^. Formation process of USIO/G is schematically shown in Fig. 9. The Fe_3_O_4_ nanoparticles with uniform distribution, which are beneficial for electrical conductivity of graphene, mitigation of volume expansion of Fe_3_O_4_, and facilitating Fe_3_O_4_ particles into the electrolyte.

Xiong et al. [128] a kind of hierarchical hollow Fe_3_O_4_ (H-Fe_3_O_4_) microspheres prepared by controlled thermal decomposition of iron alkoxide precursor. In a classical reaction, ethylene glycol (EG) serves as reduction reagent that partly reduces Fe^3+^ to Fe^2+^ with sodium acetate (NaAc), and polyvinylpyrrolidone (PVP) [128]. For this synthesis, PVP served as a surface stabilizer, which has important role in the formation and transformation of hollow interiors.

**Fig. 10:**
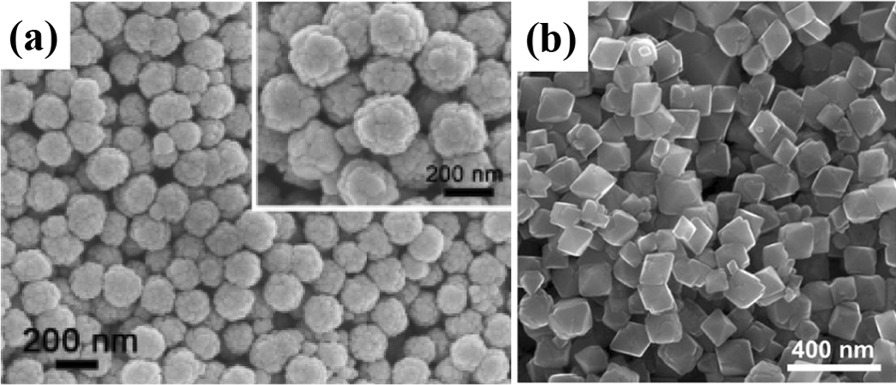
**a** SEM images of the Fe_3_O_4_ nanospheres synthesized with ethylenediamine (EDA). The inset of (**a**) shows a SEM image with higher magnification. (Reprinted with Permission from [129]. Copyright, American Chemical Society.). Physicochemical characterization of the octahedral Fe_3_O_4_ nanoparticles. **b** SEM image showing the octahedral geometry of the iron oxide nanoparticles. Reprinted with Permission from [130]. Copyright, Wiley–VCH

With the development of solvothermal synthesis, it emerging as an efficient method with the advantages of low energy consumption, little reunion and easy to control shape, etc. Chen et al. [129] synthesized poly (acrylic acid) (PAA)-entangled Fe_3_O_4_ nanospheres by a facile solvothermal method. In their synthesis, the ethylenediamine is crucial to the controlling of the uniformity of nanospheres, and the PAA molecules served as carbon source that transforms into the carbon matrix by heating treatment in inert atmosphere. As shown in SEM image of the prepared C-Fe_3_O_4_ nanospheres, very uniform spherical particles with a diameter of 150–200 nm are synthesized. Observed from SEM images in Fig. 10a, the nanospheres contain small irregular particles, and have a relatively rough surface. In the control experiment without ethylenediamine (EDA), the synthesized particles are much less uniform with a wider size distribution of 100–500 nm, allowing the formation of nanospheres with smaller size.

#### Co-precipitation

Due to its high cost-effectiveness, environmental friendliness, and facile synthesizing protocol, co-precipitation is a general approach for Fe_3_O_4_ nanoparticles. Thus, in iron based rechargeable battery systems, Fe_3_O_4_ nanomaterials are especially suitable for large-scale electrochemical applications to solve the energy requirement of the modern society.

Li et al. [121] proposed Fe_3_O_4_ polyhedron as LIBs anode for alkaline secondary batteries by a co-precipitation. Annealing temperature makes a high effect on the physical and electrochemical performance of Fe_3_O_4_ nanomaterials. The 700 °C-annealed Fe_3_O_4_ exhibited a higher electrochemical performance, such as a higher specific discharge capacity of 604.2 mA h g^−1^ with a charging efficiency of 83.9% at 120 mA g^−1^. Ooi et al. [130] demonstrated octahedral Fe_3_O_4_ nanoparticles using a facile solvothermal route. Scanning electron microscope (SEM) image of Fe_3_O_4_ nanoparticles is shown in Fig. 10b, which depicts that octahedral Fe_3_O_4_ nanoparticles with an average length of 93 ± 18 nm were prepared by the hydrothermal method, showing a roughly Gaussian size distribution. Then, the crystal structure of octahedral nanoparticles can be further evaluated by HRTEM, and the composition of the bulk sample was further characterized by XRD and X-ray photo electron spectroscopy (XPS).

#### Thermolysis

The thermolysis is small monodisperse magnetic nanocrystals synthesized by organic metal compounds in high boiling point solvents containing stabilizing agent. Previous Organic metal bodies include metal acetylacetone compounds, metal cupferron, or metal Carbonyl compounds, and usually choose fatty acids, oleic acid, or hexadecyl amine as a surfactant. Zhang et al. [120] reported ultrafine Fe_3_O_4_ nanocrystals uniformly encapsulated in two-dimensional (2D) carbon nanonetworks through thermolysis of Fe(C_5_H_7_O_2_)_3_ precursor at 350 °C under vacuum, which named as 2D Fe_3_O_4_/C nanonetworks. This facile process using low-cost precursor proposed a green approach for preparing Fe_3_O_4_/carbon composite. Additionally, compared with the reported Fe_3_O_4_/carbon composites, the particle size of Fe_3_O_4_ is controllable and a size of ∼ 3 nm can be obtained.

Benefitting from synergistic effects of carbon nanonetworks with excellent electrical conductivity and ultrafine Fe_3_O_4_ particles with uniform distribution, high reversible capacity, excellent rate capability and superior cyclability at the voltage of 0.01–3.0 V (vs. Li/Li^+^) are obtained. Nanoparticles with unique iron oxide (Fe_3_O_4_) cores and zinc oxide (ZnO) shells were prepared by Jaramillo et al. [131]. Fe_3_O_4_ nanoparticle synthesized through a thermolysis method using Fe(C_5_H_7_O_2_)_3_ as organic metal body presoma, triethylene glycol as surface active agent, and core–shell Fe_3_O_4_@ZnO nanoparticles were successfully synthesized using straightforward methodologies. The structural and optical properties of the materials were characterized using a combination of X-ray diffraction, electron microscopy, and light spectroscopy. Importantly, the purity of the core and shell phases in the Fe_3_O_4_@ZnO nanoparticles was confirmed by both XRD and TEM, and the ZnO shell was shown to increase the transparency of the core–shell nanoparticles relative to the single-component Fe_3_O_4_ nanoparticles. Zhang et al. [132] demonstrated a high crystalline Fe_3_O_4_-graphene composite by one-step reaction of thermolysis. And they demonstrated that the attachment of iron-organic complex with graphene oxide (GO) sheets can facilely result in magnetic graphene composites via a time-dependent calcination process.

#### Sol–Gel Process

The specific method is using the metal alkoxide, metal mineral compound or a mixture of the above two substances to hydrolysis and polymerization, uniform gel gradually, then condense into a transparent gel, however, after drying and heating, finally the oxide ultrafine powders was received. Tang et al. [122] prepared nanostructured magnetite thin film by sol–gel method using inexpensive iron (II) chloride precursor. Fe_3_O_4_ nanoparticles were prepared at 300 °C, however, α-Fe_2_O_3_ is generated when temperature increased to 350 °C, and this result restricts its applications. Xu et al. [123] proposed magnetite nanoparticles by virtue of sol–gel process combined with annealing in vacuum at 200–400 °C using nontoxic and low-cost ferric nitrate. In their study, Fe_3_O_4_ nanoparticles with various sizes can be synthesized facilely through varying the annealing temperature.

#### Micro-emulsion Method

Micro-emulsion is composed of two mutual miscibility of liquid mixture of thermodynamic stability and isotropy dispersion, one of these or two kinds of liquid called micro area, and fixed by interface layer of the surfactant molecules. The key factors controlling the reaction solution contain concentration, pH value, reaction time and temperature. Micro-emulsion as a rapid expansion of new technology possesses many advantages. For example, high purity and uniform particle size distribution molecular dopant was synthesized at low temperature and simple reaction process. But there are also some shortcomings, for instance, the reaction system mostly contains organic solvents, which leads to high cost, pollution of environmental health and long reaction time. The prepared Fe_3_O_4_ nanoparticles have excellent catalytic performance for the synthesis of quinoxaline in different solvents. Novel core–shell magnetic Fe_3_O_4_/silica nanocomposites with triblock-copolymer grafted on their surface (Fe_3_O_4_@SiO_2_@MDN) were successfully synthesized by combining sol–gel process with seeded aqueous-phase radical copolymerization approach [133]. The Fe_3_O_4_@SiO_2_@MDN microspheres were synthesized in following three steps. Firstly, the initial magnetic Fe_3_O_4_ microspheres were synthesized by a solvothermal reaction. Then a sol–gel process was utilized to prepare silica coated Fe_3_O_4_ microspheres (Fe_3_O_4_@SiO_2_), and a thin amorphous silica layer was formed on Fe_3_O_4_ microspheres. Afterward, the Fe_3_O_4_@SiO_2_ microspheres were modified by 3-(methacryloxypropyl) trimethoxysilane (MPS). Finally, the triblock copolymer was fabricated by aqueous phase radical copolymerization reaction among MPS, divinylbenzene (DVB) and N-Vinyl-2-pyrrolidone (NVP) on the surface of Fe_3_O_4_@SiO_2_. The magnetic Fe_3_O_4_ particles with narrow size distribution have nearly spherical shape and smooth surface. Li et al. [124] reported hexagonal and triangular monodisperse Fe_3_O_4_ nanosheets by a two-step microemulsion solvothermal approach, in which the uniform Fe_3_O_4_ nanoparticles are prepared and then these hydrophobic nanocrystals are dispersed in a uniform microemulsion environment as “seeds” for further re-growth through a secondary solvothermal process. In the first step, near-spherical monodisperse 7–8 nm Fe_3_O_4_ nanoparticles were formed through a kinetically controlled process. In the second step, the formation of anisotropic Fe_3_O_4_ nanosheets is a thermodynamically controlled process and all the exposed surfaces of the triangular and hexagonal nanosheets are (111) crystal planes, which have the lowest surface energy for FCC Fe_3_O_4_.

#### Other Methods

Physical methods are also significant ways to prepared Fe_3_O_4_ nanostructure for anode of LIBs. Several advantages, such as good crystallization, fine-tuned particle size, and high purity of products are highlighted in recent literatures. But these methods usually demand advanced and expensive equipment, result in a higher cost, poor dispensability of particles dispersion, and agglomeration of nanostructures. For instance, Du et al. [109] fabricated activated carbon (AC)-Fe_3_O_4_ nanoparticles asymmetric supercapacitor, and Fe_3_O_4_ nanostructure was prepared by microwave method. The precursor, FeSO_4_·7H_2_O and NH_3_·H_2_O mixed solution, was heated in microwave oven. The black precipitate was separated by magnet and washed repeatedly with DI water. The resulted microstructural properties of prepared nanoparticle were characterized by nitrogen adsorption (Quantachrome NOVA 2000), XRD and SEM [109]. Chen et al. [127] synthesized graphene nanosheets decorated with ultra-small Fe_3_O_4_ nanoparticles (USIO/G). Seo et al. [134] reported an integrated usage of magnetic particles in microalgal downstream processes, specifically microalgal harvesting and lipid extraction through one-step aerosol spray pyrolysis and applied in microalgal harvesting and serial microalgal lipid entrapment. TEM/EDS, XPS, and FT-IR analysis suggested that the cationic and lipophilic functionalities arose from not fully decomposed PVP, due to the short residence time in the reactor. Kang et al. [135] proposed Fe_3_O_4_ nanocrystals confined in mesocellular carbon foam (MSU-F–C) by a “host–guest” approach and applied it as LIBs anode. In this study, a precursor of Fe(NO_3_)_3_·9H_2_O is impregnated in MSU-F–C having uniform cellular pores with a diamter of ~ 30 nm, followed by heating treatment at 400 °C for 4 h in argon (Ar) atmosphere. Fe_3_O_4_ nanocrystals with size of 13–27 nm were fabricated inside the pores of MSU-F–C. The existance of the carbon most likely allows the reduction of some Fe^3+^ to Fe^2+^ ions by a carbothermoreduction process. The physical performance and pore structure of MSU-F–C and Fe_3_O_4_-loaded composites were characterized with nitrogen sorption, and the composites have high capacities of ∼800–1000 mA h g^−1^ at 0.1 A g^−1^ (∼0.1 C rate), high rate capability and good cycling performance.

### Application

Fe_3_O_4_ possesses lots of unique properties, and is highly promising for applications in LIBs and supercapacitors [136–142]. Table 4 summarizes some applications.

**Table 4:** A Summary of synthesis and electrochemical performance of Fe_3_O_4_ based nanostructures

Structure	Synthesis	Electrochemical performance	Ref
Fe_3_O_4_ nanoparticles	Hydrothermal synthesis	Capacitance of 207.7 F g^−1^ at 0.4 A g^−1^; retention of 100% after 2000 cycles	[106]
Hierarchical porous Fe_3_O_4_/graphene nanowires	Hydrothermal synthesis	Capacity of 1146 mA h g^−1^ at 5 A g^−1^	[51]
Fe_3_O_4_ particles/carbon nanonetworks	Thermolysis	Capacity of 1534 mA h g^−1^ at a 1 C; without decay up to 500 cycles (1 C = 1 A g^−1^)	[109]
Fe_3_O_4_ doped double-shelled hollow carbon spheres	Rapid aerosol	Capacitance of 1153 F g^−1^ at 2 A g^−1^; retention of 96.7% after 8000 cycles	[4]

#### Li-Ion Batteries

**Fig. 11:**
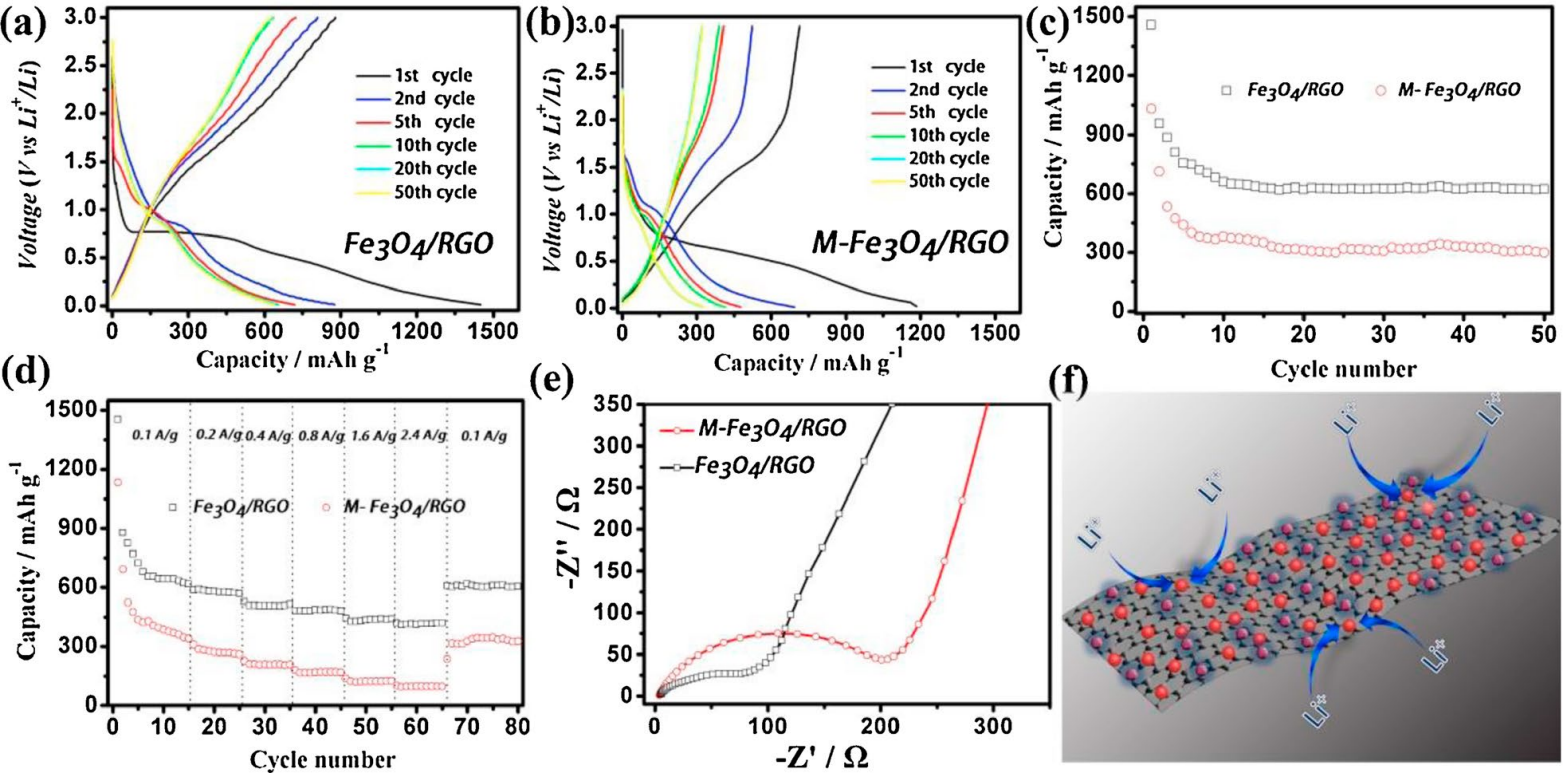
The charge/discharge curve of Fe_3_O_4_/RGO composites (**a**) and mechanically mixed Fe_3_O_4_/RGO composites (M-Fe_3_O_4_/RGO) (**b**) electrodes at constant current densities of 0.1 A g^−1^. Cycling performance of Fe_3_O_4_/RGO composites and M-Fe_3_O_4_/RGO composites electrode at constant current densities of 0.1 A g^−1^ (**c**). Rate capability of Fe_3_O_4/_RGO composites and physically mixed Fe_3_O_4_/RGO composites at the current densities between 0.1 A g^−1^ and 2.4 A g^−1^ (**d**). Nyquist plots of the electrodes of Fe_3_O_4_/RGO sheet and mechanically mixed Fe_3_O_4_/RGO composites. All of the measurements were conducted using a voltage window of 0.01–3.0 V (**e**). Schematic representation of the electrochemical reaction path on the Fe_3_O_4_/RGO composites (**f**). Reprinted with Permission from [3]. Copyright, Elsevier B.V

Due to conversion reaction of Fe_3_O_4_ during charge/discharge process and other advantages, the Fe_3_O_4_ is usually studied and applied as LIBs anode [143–147]. For TMOs, they have higher theoretical capacity (~ 500–1000 mA h g^−1^) than conventional graphite (about 372 mA h g^−1^). Furthermore, Fe_3_O_4_ has superior conductivity compared with other transition metal oxides. Thus, it is well-focused by recent studies. It has been reported that composite electrodes with graphene have high performance due to their large surface area, high electrical conductivity and adaptive or flexible structure for high reliability. Qiu et al. [3] reported a kind of composite anode composed of ultra-dispersed Fe_3_O_4_ nanoparticles (3–8 nm) and RGO sheet. It has excellent cyclic performance (624 mA h g^−1^ for up to 50 charge/discharge cycles at a current density of 0.1 A g^−1^), and good specific capability (624 and 415 mAh g^−1^ at 0.1 and 2.4 A g^−1^, respectively) for LIBs. The obtained Fe_3_O_4_/RGO exhibited high and ultrastable Photo-Fenton activity (Fig. 11).

**Fig. 12:**
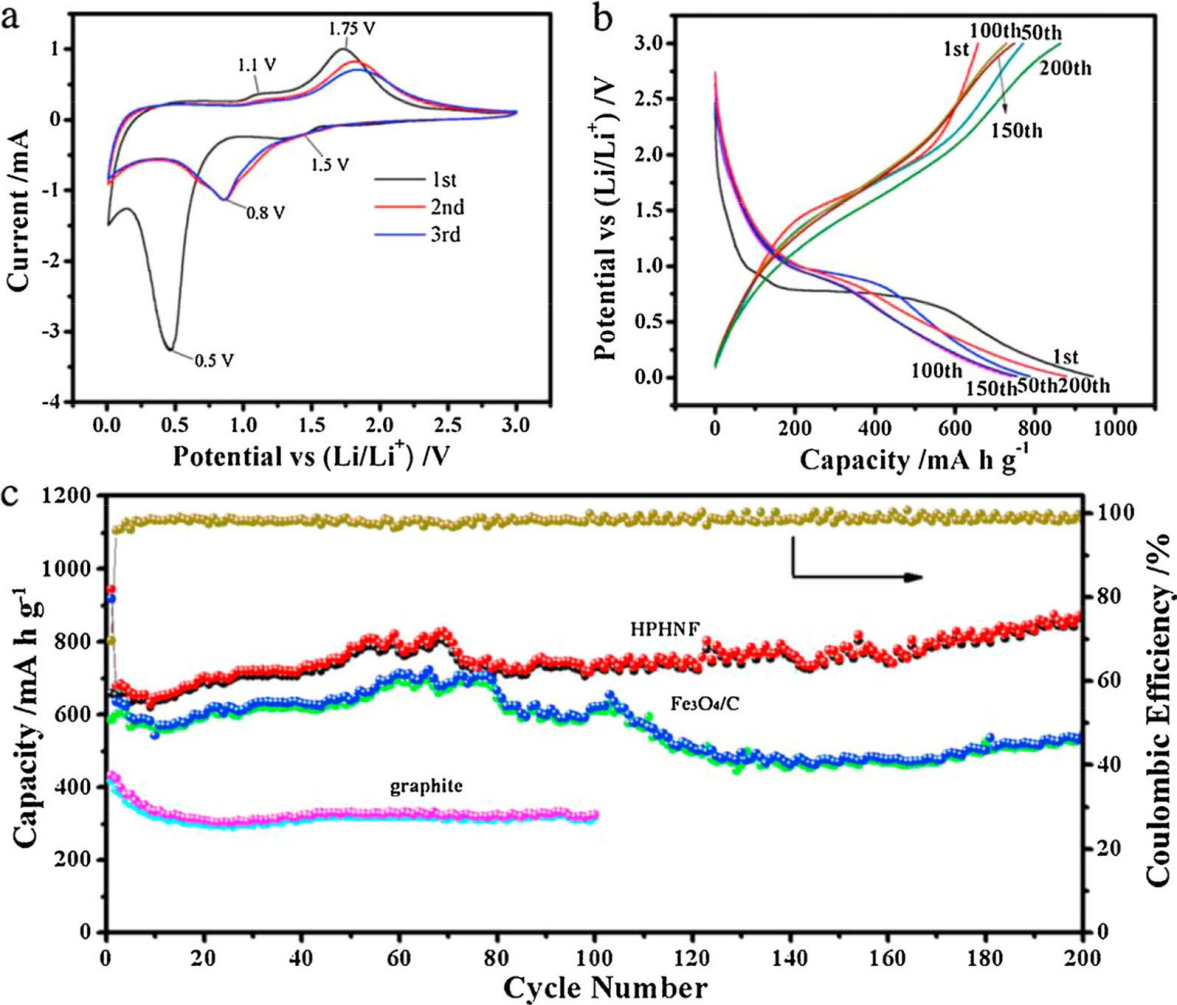
**a** CVs of the HPHNF during the first three cycles at 0.2 mV s^−1^, **b** Galvanostatic charge/discharge profiles of the HPHNF electrodes for the 1st, 50th, 100th, 150th and 200th cycle at a specific current of 200 mA g^−1^. **c** Cycling performance of the HPHNF nanocomposites, N-doped Fe_3_O_4_/C nanocomposites and graphite at a specific current of 200 mA g^−1^. **d** Coulombic efficiency of HPHNF. Reprinted with Permission from [12], Copyright, Elsevier Ltd

Pyrolyzed carbon is also a good “companion” for Fe_3_O_4_ anodes. Apart from the facile protocol, porous structure formed by pyrolysis always exhibits high specific capacity of Fe_3_O_4_ composite anode. Wang et al. [12] reported hollow N-doped Fe_3_O_4_/C nanocages with hierarchical porosities by carbonizing polydopamine-coated PB nanocrystals as LIBs anode (Fig. 12). The specific capacity of N-doped Fe_3_O_4_/C nanocages is ~ 878.7 mA h g^−1^ after 200 cycles at a current density of 200 mA g^−1^, much higher than that of N-doped Fe_3_O_4_/C derived from pure PB (merely 547 mA h g^−1^). It is also desirable to design anisotropic structure of Fe_3_O_4_ nanoparticles with carbon coated layer. Zhang et al. [19] reported a kind of carbon-coated Fe_3_O_4_ nanospindles derived from α-Fe_2_O_3_ nanospindles with length of about 500 nm and an axis ratio of ~ 4. Following by a hydrothermal synthesis method with glucose, the obtained LIBs anode delivered a high reversible capacity of ~ 745 mA h g^−1^ at C/5 and ~ 600 mA h g^−1^ at C/2.

The most impressive work towards this field is probably the mesoporous iron oxide nanoparticle clusters with carbon coating reported by Lee et al. [148]. After a few cycles, the formation of SEI greatly enhanced the stability of interface between electrode and electrolyte. Electrochemical test exhibited a high specific capacity of 970 mA h g^−1^ for LIBs.

#### Supercapacitors

Fe_3_O_4_ is a highly promising candidate for supercapacitor electrode because of its relatively high electrical conductivity, fast reversible redox reaction, low cost and eco-friendly nature [149–152]. Similar to batteries, high performance supercapacitors also require two factors: large specific surface area and long-term stability. Those two features usually were achieved by building some porous structures and carbon coated layers. Fe_3_O_4_ nanoparticle with a high specific surface area was synthesized by Wang et al. [126] using a bottom up approach. Ferric chloride was firstly sonicated with ethanolamine and then processed through a solvothermal reaction. The obtained active nanomaterials showed a specific surface area of 165.05 m^2^ g^−1^ and a specific capacitance of 207.7 F g^−1^ at 0.4 A g^−1^.

Also, highly dispersed Fe_3_O_4_ nanosheets on 1D CNFs is reported by Mu et al. [125]. The Fe_3_O_4_/CNFs composites showed a higher specific capacitance than pure Fe_3_O_4_ in 1 M Na_2_SO_3_. To further enlarge the specific capacitance and cycle stability, hierarchically porous carbon spheres with Fe_3_O_4_ using as supercapacitors exhibited high capacitivity of 1153 F g^−1^ at 2 A g^−1^ and high specific capacitance of 514 F g^−1^ at 100 A g^−1^. In addition, the assembled asymmetric supercapacitor with double-shelled hollow carbon spheres and Fe_3_O_4_, has excellent cycling stability (96.7% retention after 8000 cycles) and high energy density (17–45 Wh kg^−1^) at a power density of 400–8000 W kg^−1^ [4].

## Conclusion

Iron oxides (Fe_1−*x*_O, Fe_2_O_3_, Fe_3_O_4_) based nanostructures have much higher specific capacities than those of commercial carbon based anodes. They are considered as highly promising candidates for LIBs anode. However, large irreversible capacity and low cycle stability are two serious problems that obstruct the application of iron oxides based nanostructures. In this review, we summarized the recent progress on novel iron oxides and their composites as LIBs anode and supercapacitor electrode. Several typical synthetic methods of various novel iron oxides based nanostructures are listed. By comparing the electrochemical performance of these various iron oxides based nanostructures, some strategies are expected to solve the problems of iron oxides based nanostructures.

## Data Availability

Not applicable.
